# Influence of cyclodextrin on the solubility of a classically prepared 2-vinylcyclopropane macromonomer in aqueous solution

**DOI:** 10.3762/bjoc.8.173

**Published:** 2012-09-13

**Authors:** Helmut Ritter, Jia Cheng, Monir Tabatabai

**Affiliations:** 1 Heinrich-Heine-Universität Düsseldorf, Institut für Organische Chemie und Macromolekulare Chemie, Universitätsstraße 1, 40225 Düsseldorf, Germany, Fax: (+49) 211-811-5840

**Keywords:** branched poly(NiPAAm), cloud point, cyclodextrins, graft copolymer, macromonomer, ring-opening free radical polymerization, 2-vinylcyclopropane

## Abstract

A macromonomer **5** consisting of a polymerizable vinylcyclopropane end group and a poly(*N*-isopropylacrylamide) (poly(NiPAAm)) chain was obtained from amidation of 1-ethoxycarbonyl-2-vinylcyclopropane-1-carboxylic acid (**4**) with an amino-terminated poly(NiPAAm) **3** as an example. This macromonomer **5** showed an LCST effect after complexation of the vinyl end group with ß-cyclodextrin in water. Via radical ring-opening copolymerization of **5** and NiPAAm a graft copolymer **8** with a clouding point of 32 °C was synthesized. The branched unsaturated polymer was treated with ozone to cleave the double bonds of the main chain.

## Introduction

Macromonomers are polymers or oligomers with at least one functional end group that is capable of further polymerization. The molecular weight of macromonomers generally ranges between 1000 and 20000 g/mol [1]. In the last decades, a considerable amount of studies on the synthesis and applications of macromonomers has been reported [2–5]. They enable easy and direct synthesis of a variety of graft copolymers [6], which consist of a linear main chain and randomly distributed side chains. Graft copolymers have found many applications, for example in the field of coatings, adhesives, compatibilizers, emulsifiers or biomaterials [1]. For the synthesis of graft copolymers, free radical copolymerization of macromonomers with suitable low molecular weight vinylmonomers is a widely studied area [7–8].

β-Cyclodextrins (β-CDs) are cyclic oligomers consisting of seven α-1,4-glycosidically linked glucopyranose units which are present in the chair conformation [9–12]. The molecule resembles a truncated cone with approximate *C*_n_-symmetry [13]. The cyclic 1,4-linked glucose units in the molecule arrange themselves in a way that a hydrophilic exterior and a hydrophobic interior are created [14–16] and β-CDs are easily dissoluble in polar solvents such as water. The interior height of β-CDs is 7.9 Å and the interior width is between 6.0–6.5 Å, which allows an inclusion of different types of molecules with fitting size through van-der-Waals interactions. By formation of such host–guest complex properties, the dissolution behavior of the guest molecules can be changed. Based on this fact numerous applications of β-CDs are reported [17–25].

**Scheme 1:**
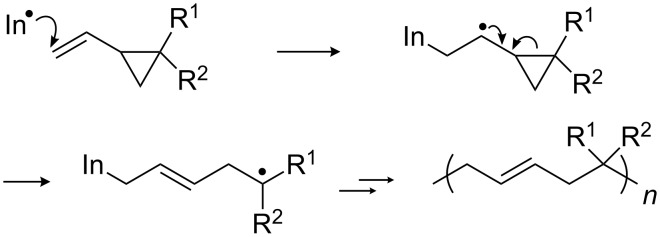
Mechanism of free radical ring-opening polymerization of 2-VCPs (In: initiator) [29–31].

Generally, 2-vinylcyclopropane monomers (2-VCPs) are known for their low volumetric shrinkage or even small expansion during free radical ring-opening polymerization (Scheme 1) [26–28]. However, this behavior is not in focus of the present work. The resulting polymer bears mainly 1,5-ring-opened units with a partially unsaturated backbone [29–31], which is suitable for further modifications.

As mentioned above, polymerization reactions and mechanical and chemical modifications of vinylcyclopropane derivates have been carefully investigated in recent years. However, up to now, nothing is known in literature about the behavior of vinylcyclopropane-containing macromonomers. Thus, in this paper we are going to report our findings about the preparation and characterization of a new type of macromonomers based on a 2-vinylcyclopropane derivative bearing a thermoresponsive poly(NiPAAm) moiety as an example, with the intention to demonstrate the great potentials of this class of 2-vinylcyclopropane monomers. Additionally, the degradation of unsaturated poly(vinylcyclopropan) backbones using ozone is first reported.

## Results and Discussion

**Scheme 2:**
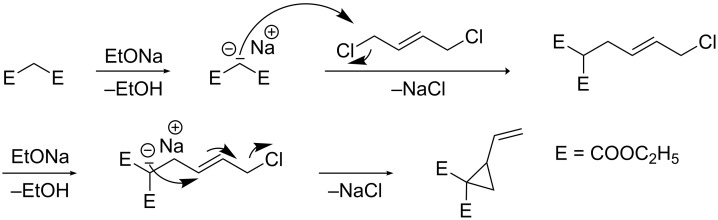
Synthesis of diethyl 2-vinyl-1,1-cyclopropanedicarboxylate [33].

1-Ethoxycarbonyl-2-vinylcyclopropane-1-carboxylic acid (**4**) was synthesized following a procedure described in [32]. Diethyl 2-vinyl-1,1-cyclopropanedicarboxylate could be obtained according to [33] from sodium ethoxide, diethyl malonate and *trans*-1,4-dichloro-2-butene in ethanol (Scheme 2).

**Scheme 3:**
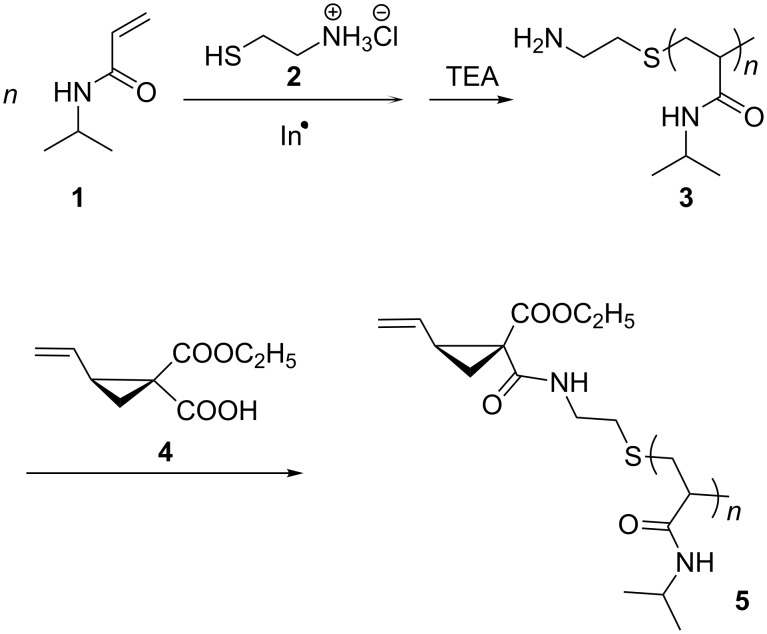
Two-step synthesis of the macromonomer **5** (In: Initiator, TEA: triethylamine).

The desired macromonomer **5** was prepared in two steps according to Scheme 3. The intermediate amino-terminated poly(NiPAAm) **3** was obtained from the reaction of NiPAAm (**1**) and 2-aminoethanethiol hydrochloride (**2**) via radical polymerization either in water using **2** and ammonium persulfate as redox initiator [34] or in ethanol using 2,2’-azobis(isobutyronitrile) (AIBN) as radical initiator and **2** as chain transfer agent. The implementation in aqueous solution represents an environmentally friendly alternative to conventional polymerization. To avoid the Michael addition between the amino group and the acryloyl group, the ammonium salt of **2** was used instead of uncharged nucleophilic 2-aminoethanethiol. After treating with triethylamine, amino-terminated polymer **3** was obtained. The amidation of the amino group of **3** with 1-ethoxycarbonyl-2-vinylcyclopropane-1-carboxylic acid (**4**) in the presence of *N,N*′-dicyclohexylcarbodiimide (DCC) as condensation agent led to the formation of macromonomer **5**.

**Figure 1:**
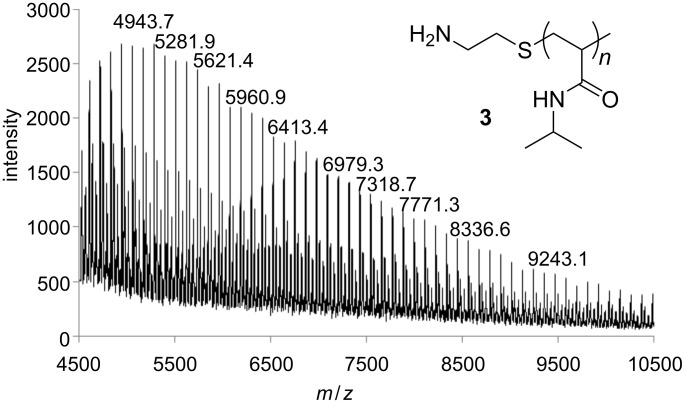
MALDI-TOF MS of amino-terminated poly(NiPAAm) **3**.

**Figure 2:**
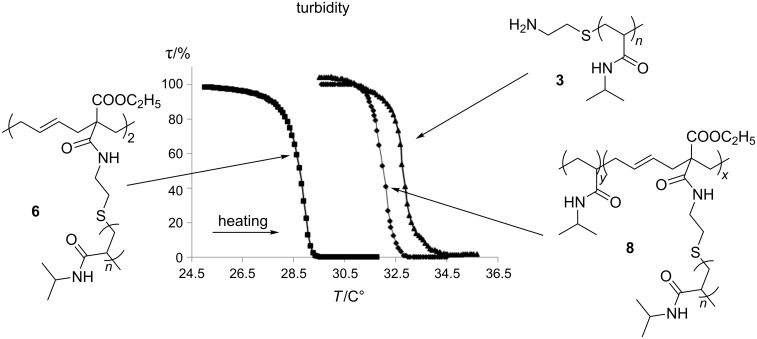
Optical transmittance of aqueous solutions (*c* = 20 mg/mL) of **3**, **6** und **8** during heating.

Compared to the FTIR spectrum of NiPAAm (**1**), the spectrum of **3** shows the disappearance of the C=C bonds band at 1619 cm^−1^ and the appearance of a new band of primary amino groups at 3436 cm^−1^. In the ^1^H NMR spectrum, the chemical shifts of the methylene protons adjacent to the amino group at δ 2.85 ppm and the methylene protons adjacent to the thio group at δ 2.68 ppm which also confirms the structure of **3**. The mass differences between the signals of the main series in MALDI-TOF MS correspond to the molar mass of the monomer NiPAAm units equal to 113 g/mol and the mass of end group agrees with the molar mass of 2-aminoethanethiol (77 g/mol, Figure 1), as expected. The number average molecular weight (*M*_n_) and the dispersity index (DI) of **3** were measured with GPC and were 10300 g/mol and 2.8, respectively (Table 1). The hydrodynamic diameter (*d*_n_) of **3** determined by means of DLS was about 4.1 nm. DSC measurement showed the glass-transition temperature (*T*_g_) at about 123.5 °C. Due to the hydrophilicity of the amino end groups of **3** a typical LCST (lower critical solution temperature) was observed close to that of unmodified poly(NiPAAm) at about 33.4 °C (Figure 2).

**Table 1:** Properties of synthesized macromonomers and polymers.

	*M*_n_ (10^4^ g/mol)	DI	*d*_n_^a^ (nm)	cloud point^b^ (°C)	*T*_g_ (°C)	yield (%)

**3**	1.0	2.8	4.1	33.4	123.5	54^c^ or 66^d^
**5**	1.2	2.5	5.0	–	129.1	86
**7**			–	31.7		–
**6**	2.3	1.7	5.6	29.9	135.3	53
**8**	4.8	2.5	6.4	32.2	133.7	65

^a^5 mg/mL in acetone at 25 °C; ^b^20 mg/mL in water; ^c^polymerization in water; ^d^polymerization in ethanol.

Via condensation of the amino end group of **3** with the carboxyl group of **4** the corresponding macromonomer **5** with *M*_n_ = 11540 g/mol und *T*_g_ = 129.1 °C was obtained. The ^1^H NMR spectrum confirms the existence of a vinylcyclopropane unit by the presence of the multiplets at δ 1.48–1.61 ppm and δ 4.98–5.19 ppm, assignable to the CH of the cyclopropane ring and of the vinyl group, respectively. The FTIR spectrum shows the typical absorption band of ester groups at 1726 cm^−1^ and the disappearance of the band of primary amino groups; this also proves the successful condensation between **3** and **4**. Due to the faintly voluminous vinylcyclopropane unit **5** possesses a slightly larger *d*_n_ than **3** (by ca. 0.9 nm, Table 1).

**Figure 3:**
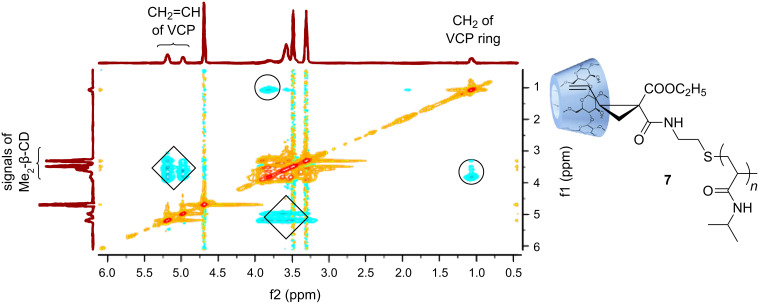
2D ROESY NMR spectrum of a **5**/Me_2_-β-CD deuterated water solution.

**Figure 4:**
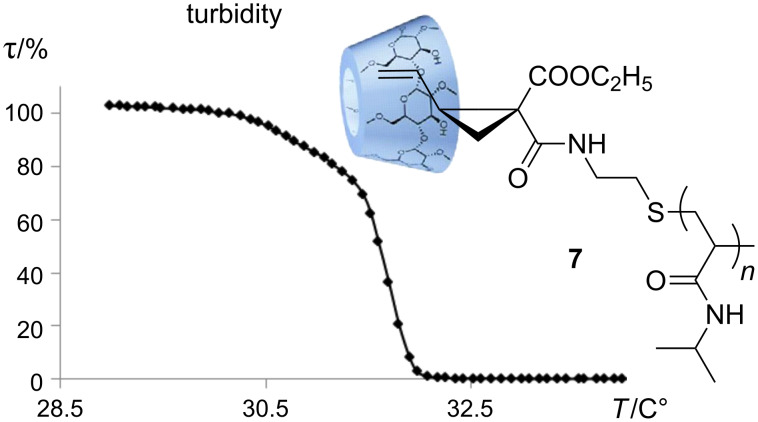
Temperature-dependent transparency measurements of aqueous solution of the supramolecular complex **7** (*c* = 20 mg/mL in water).

In general, the cloud point of poly(NiPAAm) with relative low molecular weight can be influenced by the structure of end groups. The relatively hydrophobic vinylcyclopropane end group hampers the aqueous solubility of **5** at room temperature (about 25 °C). However, the turbid dispersion becomes completely clear by the addition of methylated β-cyclodextrin (Me_2_-β-CD). This means that a water-soluble inclusion complex **7** of **5** with Me_2_-β-CD is formed (Figure 3). The 2D ROESY NMR spectrum of **7** indicates the noncovalent interaction between the Me_2_-β-CD ring and the vinyl end group of macromonomer **5** (Figure 2). It can be noticed that the protons of the vinyl groups (CH_2_=CH) at δ 4.98–5.19 ppm and the protons of the cyclopropan units at δ 1.48–1.61 ppm correlated to the protons of Me_2_-β-CD at δ 3.3–3.9 ppm. Therefore, we came to the conclusion that the Me_2_-β-CD ring preferably includes the vinylcyclopropane unit instead of the isopropyl unit. The supramolecular complex **7** shows the typical LCST behavior (31.7 °C) of poly(NiPAAm) (Figure 4).

**Scheme 4:**
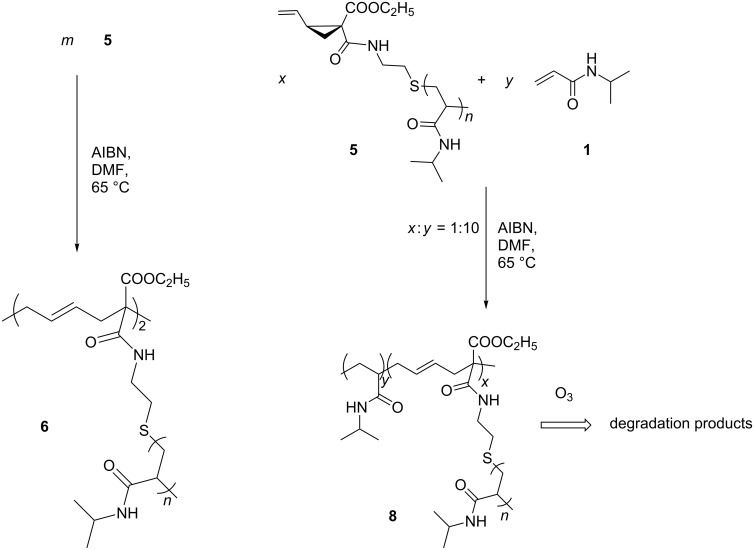
Homo- and copolymerization of macromonomer **5**.

After free radical initiated ring-opening polymerization of **5**, a product **6** was formed (Scheme 4). However, GPC of **6** shows only a preferred dimerization of macromonomer **5**, which may result from the relative bulky poly(NiPAAm) side chains. Therefore, copolymerization of **5** and NiPAAm was carried out. In this case, as expected, a copolymer **8** was obtained with much higher *M*_n_ value of 48300 g/mol according to GPC measurement. The disappearance of NMR-signals of the vinylcyclopropane unit of **5** at δ 4.98–5.19 ppm and the appearance of a new peak at δ 5.6 ppm, which is assigned to the protons of CH=CH in the poly(vinylcyclopropane) main chain, prove the ring-opening polymerization of **5**. In addition, the product has only shown one glass-transition temperature *T*_g_ at 133.7 °C. This confirmed the formation of a copolymer **8**. If two homopolymers coexist, two glass-transition temperatures should be measured. Both products **6** and **8** show the LCST behavior, respectively (Figure 2). After ozonolytic degradation of **8** in methanol, the disappearance of the protons of the double bond signal at δ 5.6 ppm in ^1^H NMR spectrum and the appearance of the aldehyde protons at δ 8.5 ppm evidence the effective degradation of copolymer. The *M*_n_ of the degradation products amounts to 1.8 × 10^4^ g/mol with DI equal to 2.2. Altogether, the successful ozonolytic degradation is proved.

## Conclusion

A new macromonomer **5** was synthesized via amidation of 1-ethoxycarbonyl-2-vinylcyclopropane-1-carboxylic acid (**4**) with an amino-terminated poly(NiPAAm) **3** as an example. Thanks to its relative hydrophobic vinylcyclopropane unit, this water insoluble macromonomer **5** is able to form a host–guest complex with Me_2_-β-CD, which is located at the polymerizable vinyl end group. This supramolecular complex **7** becomes completely water-soluble, so that a typical LCST effect could be observed. Since the bulky macromonomer **5** prefers to form only dimers in presence of a radical initiator rather than a homopolymer, a graft copolymer **8** was formed via radical ring-opening copolymerization of **5** and NiPAAm. The double bonds of the main chain can be cleaved simply by ozone treatment.

## Experimental

All chemicals were commercially available and used as received without further purification. All solvents were distilled and dried over molecular sieve before use. ATR-FTIR-spectra were recorded with an FTIR-5SXB by Nicolet at room temperature. NMR spectra were recorded with an AVIII-300 instrument at 300 MHz at 25 °C. Tetramethylsilane (TMS) was used to calibrate the δ-scale. Chemical shifts were referenced to the residual solvent peaks (for example δ 2.09 ppm for acetone-*d*_6_ and δ 2.22 ppm for D_2_O). Dynamic light scattering (DLS) measurements were performed on a Malvern high performance particle sizer-extended temperature (HPPS-ET) instrument equipped with a He–Ne-laser and an Avalanche photodiode detector. The turbidity measurements were carried out using a power-regulated semiconductor laser (λ = 670 nm) and a silicon photodiode in a TP1 turbidity photometer from TEPPER-Analytik. Glass-transition temperatures (*T*_g_) were measured using differential scanning calorimetry (DSC) with a Perkin-Elmer Model DSC-7 in nitrogen atmosphere; about 10 mg samples were used at a scan rate of 15 K/min. *T*_g_ was taken as the average of three measurements using the midpoint method. LCST were determined by transmission changes (at 500 nm) of the solutions heated at 1 °C/min^−1^ in a magnetically stirred cell; values of the cloud points were defined as the temperature at which the deepest point of the derivative curve was achieved. MALDI-TOF MS was performed on a Bruker Ultraflex TDF mass spectrometer using a 337 nm nitrogen laser. GPC analyses were performed with a Viscotek GPC_max_ VE2001 using DMF as the eluent at 60 °C (flow rate: 1 mL/min). The ozonolysis was carried out on an ozone generator by Fischer Technology. In this ozone generator 45–50 liters of oxygen per hour was provided.

### Synthesis of amino-terminated poly(*N*-isopropylacrylamide) **3**

A) In water: An aqueous solution of NiPAAm (12.45 g, 110 mmol) and 2-aminoethanethiol hydrochloride (0.31 g, 2.7 mmol) was deaerated with nitrogen bubbling for 1 h, and then, a 40 wt % aqueous solution of ammonium persulfate (0.62 g, 2.7 mmol) was added. The homogenous solution was stirred at room temperature (about 25 °C) for 4 h. After that, 1 mL Et_3_N was dropped into the solution. Then, the solution was warmed to 40 °C and the polymer **3** precipitated thereby completely because of LCST. After filtration, washing with hot water for several times and drying, the polymer **3** (6.66 g, yield 54%) was obtained as a white powder.

B) In ethanol: A solution of NiPAAm (12.45 g, 110 mmol) and 2-aminoethanethiol hydrochloride (0.31 g, 2.7 mmol) in anhydrous ethanol (100 mL) was deaerated with nitrogen bubbling for 1 h. Subsequently, a solution of AIBN (54 mg, 0.33 mmol) in anhydrous ethanol (1 mL) was added. Afterwards, the solution was stirred at 65 °C for 12 h. Then, 1 mL of Et_3_N was dropped into the solution. After evaporation of ethanol in vacuo the solid was dissolved in acetone. The precipitate formed was removed by filtration. The polymer **3** (8.17 g, yield 66%) was obtained as a white powder after it had been precipitated from ethanol in hexane, filtered off and dried.

FTIR (diamond) 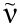 (cm^−1^): 3436 (ν NH of NH_2_), 3280 (ν NH of amide), 2970–2928 (ν CH aliphatic), 1655 (ν C=O, amide I), 1535 (δ NH, amide II); ^1^H NMR (D_2_O, 300 MHz) δ (ppm) 3.86 (*CH*NH), 2.85 (C*H_2_*NH_2_), 2.68 (CH_2_C*H_2_*S), 2.47 (*CH*CO), 2.10 (SC*H_2_*CH), 1.41–1.01 (C*H_2_*, C*H_3_*).

### Synthesis of macromonomer **5**

Ethoxycarbonyl-2-vinylcyclopropane-1-carboxylic acid (**4**) was synthesized following a procedure described in [32]. A solution of **3** (2 g) and **4** (0.5 g, 2.7 mmol) in 50 mL of anhydrous dichlormethane in a one-necked flask equipped with a calcium chloride drying tube was cooled to 0–5 °C. Subsequently, DCC (0.56 g, 2.7 mmol) in 2 mL anhydrous dichlormethane was slowly added. After a further 30 min at 0–5 °C the mixture was stirred for 24 h at room temperature. The precipitated dicyclohexylurea was filtered off and the organic solution was washed with three 20 mL portions of 5% HCl, three 20 mL portions of saturated NaHCO_3_ solution and three 20 mL portions of water and then dried over Na_2_SO_4_. After precipitation from dichlormethane in hexane, the macromonomer **5** (1.72 g, yield 86%) was obtained as a white powder. FTIR (diamond) 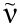 (cm^−1^): 3280 (ν NH), 2970–2930 (ν CH aliphatic), 1726 (ν C=O of ester), 1639 (ν C=O, amide I), 1545 (δ NH, amide II); ^1^H NMR (acetone-*d*_6_, 300 MHz) δ (ppm) 7.13 (N*H*), 5.19–4.98 (C*H_2_*=C*H*), 4.11 (COOC*H_2_*), 3.85 (*CH*NH), 2.99 (C*H* of cyclopropane), 2.82 (C*H_2_*NH_2_), 2.68 (CH_2_C*H_2_*S), 2.47 (*CH*CO), 2.10 (SC*H_2_*CH), 1.61–1.48 (C*H_2_* of cyclopropane), 1.40–1.01 (C*H_2_*, C*H_3_*).

### Oligomerization of macromonomer **5** to **6**

A solution of **5** (0.7 g) in anhydrous DMF (50 mL) was deaerated with nitrogen bubbling for 1 h. Subsequently, a solution of AIBN (0.42 mg, 2.56 µmol) in anhydrous DMF (0.5 mL) was added. The polymerization was carried out at 65 °C for 12 h. The synthesized polymer **6** was purified by precipitation from DMF into diethylether. An amount of 0.37 g of **6** (yield 53%) was obtained as a white powder. FTIR (diamond) 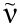 (cm^−1^): 3291 (ν NH), 2970–2930 (υ CH aliphatic), 1726 (ν C=O of ester), 1636 (ν C=O, amide I), 1541 (δ NH, amide II); ^1^H NMR (D_2_O, 300 MHz) δ (ppm) 5.5 (C*H*=C*H*), 4.01 (COOC*H_2_*), 3.80 (*CH*NH), 2.88 (C*H_2_*NH_2_), 2.70 (CH_2_C*H_2_*S), 2.52 (*CH*CO), 2.28 (SC*H_2_*CH), 1.45–0.95 (C*H_2_*, C*H_3_*).

### Copolymerization of macromonomer **5** with NiPAAm to **8**

A solution of **5** (0.7 g, 0.06 mmol) and NiPAAm (68.6 mg, 0.6 mmol) in anhydrous DMF (10 mL) was deaerated with nitrogen bubbling for 1 h. Subsequently, a solution of AIBN (0.2 mg, 1.32 µmol) in anhydrous DMF (0.2 mL) was added. The polymerization was carried out at 65 °C for 24 h. After removal of the solvent under reduced pressure the polymeric material was dissolved in water, dialyzed for three days at rt and then freeze-dried. An amount of 0.5 g of **8** (yield 65.1%) was obtained. FTIR (diamond) 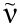 (cm^−1^): 3331 (ν NH), 2970–2930 (ν CH aliphatic), 1728 (ν C=O of ester), 1655 (ν C=O, amide I), 1561 (δ NH, amide II); ^1^H NMR (D_2_O, 300 MHz) δ (ppm) 5.6 (C*H*=C*H*), 4.21 (COOC*H_2_*), 3.85 (*CH*NH), 2.89 (C*H_2_*NH_2_), 2.72 (CH_2_C*H_2_*S), 2.55 (*CH*CO), 2.28 (SC*H_2_*CH), 1.55–1.05 (C*H_2_*, C*H_3_*).

### Complex formation of **5** with Me_2_-β-CD

Compound **5** (100 mg) was dispersed in 5 mL of distilled water at rt. While stirring, Me_2_-β-CD was gradually added until a clear solution was obtained. Then, the amount of Me_2_-β-CD that has been used was calculated; in this case ca. 500 mg, corresponding [Me_2_-β-CD]/[**5**] ratio of about 0.02:1.

### Ozonolysis of copolymer **8**

A condensation trap with 1 g of copolymer **8** is filled up with methanol until the inlet is immersed in the solution. The cold trap is connected with the ozone generator and flushed with nitrogen for 10 min. Then, the solution is cooled to −74 °C and finally flushed with oxygen. After starting the ozone generator the oxygen is converted to ozone. The ozonolysis continues until the solution turns blue due to the excess of ozone. After shutting off the ozone generator, the solution is flushed with nitrogen until it is colorless. Then, 4 mL of dimethyl sulfide (DMS) are added to the solution. The reaction mixture is stirred overnight at rt. Methanol is removed under reduced pressure. *M*_n_ = 1.8 × 10^4^ g/mol, DI = 2.2; *d*_n_ = 5.2 nm.
